# Ischemic Heart Disease and the Epidemiologic Transition: Progress without Reduction in Global Burden

**DOI:** 10.15190/d.2026.5

**Published:** 2026-03-31

**Authors:** Nahui Samanta Nájera-Segura, Zoila Mora Guzmán, Sonia Moreno-Cabral, María Magdalena Serrano Ortega, César Zárate-Ortiz, Laura Pérez-Campos Mayoral, Efrén Emmanuel Jarquín González, Guillermo Barreto, Eduardo Pérez-Campos, Hector Alejandro Cabrera-Fuentes, María Teresa Hernández-Huerta, Victor Serebruany

**Affiliations:** ^1^Centro de Investigación Facultad de Medicina UNAM-UABJO, Facultad de Medicina y Cirugía, Universidad Autónoma "Benito Juárez" de Oaxaca (UABJO), Oaxaca, México; ^2^División de Estudios de Posgrado e Investigación, Tecnológico Nacional de México, Instituto Tecnológico del Valle de Etla, Santiago Suchilquitongo, Oaxaca, México; ^3^División de Estudios de Posgrado e Investigación, Tecnológico Nacional de México / Instituto Tecnológico de Tijuana, Tijuana, Baja California, México; ^4^Laboratorio de Bioquímica de Proteínas y Glicopatologías, Facultad de Odontología, Universidad Autónoma Benito Juárez de Oaxaca (UABJO), Oaxaca, México; ^5^Dirección General de los Servicios de Salud de Oaxaca, Secretaria de Salud, Servicios de Salud de Oaxaca, Oaxaca, Mexico; ^6^Université de Lorraine, CNRS, Laboratoire IMoPA, UMR 7365, Nancy, France; ^7^División de Estudios de Posgrado e Investigación, Tecnológico Nacional de México / Instituto Tecnológico Oaxaca, Oaxaca, México; ^8^SECIHTI, Facultad de Medicina y Cirugía, Universidad Autónoma "Benito Juárez" de Oaxaca (UABJO), Oaxaca, Mexico; ^9^HeartDrug Research LLC, West Friendship, MD, USA; ^10^Department of Neurology, Johns Hopkins University School of Medicine, Baltimore, MD, USA

**Keywords:** Ischemic heart disease (IHD), Global burden of disease (GBD), Health equity, Epidemiologic transition, Cardiovascular prevention, Implementation science, Early-onset cardiovascular disease, Health systems.

## Abstract

Ischemic heart disease (IHD) remains the leading cause of cardiovascular mortality worldwide. Although age standardized death rates have declined over the past two decades, the absolute number of deaths continues to rise due to population growth and demographic aging. This Perspective examines the resulting paradox of progress, in which improving mortality rates coexist with an expanding global burden. Emerging evidence from recent global analyses highlights widening disparities across regions, sexes, and age groups. Global Burden of Disease (GBD) studies suggest an increasing burden of early onset IHD among adults aged 15 to 49 years, associated with rising incidence and prevalence, with notable regional variability and links to metabolic and dietary risks. While high income settings continue to achieve sustained mortality reductions, low- and middle-income regions face persistent gaps in prevention and care. These disparities reflect differences in health system capacity, including limited screening, delayed access to acute cardiac care, and suboptimal use of secondary prevention. Scalable strategies such as task-sharing and simplified treatment approaches offer practical solutions but remain underused. A strategic shift toward implementation, life-course prevention, and equity-focused policy reform is essential. Importantly, this perspective bridges the gap between epidemiological trends and health policy, linking epidemiologic trends to scalable implementation strategies for clinicians and policymakers to address the global burden of IHD.

## SUMMARY

*1. Introduction*

*2. Advances in Global Epidemiology and Measurement*

- *2.1. Refinement of Mortality Surveillance*

- *2.2. Integration of Risk Attribution*

*3. Regional Inequities and Health System Determinants*

*4. Sex and Age-Specific Patterns: Beyond Biology*

- *4.1. Persistent Sex Differences and the Burden of the Young*

- *4.2. Aging Populations and the Absolute Burden*

*5. Limitations of Current Epidemiological Models*

*6. Future Directions and Policy Imperatives*

- *6.1. Strengthening Global Surveillance and Data Systems*

- *6.2. Achieving Equitable Prevention and Clinical Care*

- *6.3. Implementing Sex and Age Responsive Health Policies*

- *6.4. Prioritizing Implementation Science and Policy Reform*

*7. Conclusion: From Metrics to Mortality*

## 1. Introduction

Ischemic heart disease (IHD) has long occupied a central position in global health, accounting for more deaths annually than any other cardiovascular condition^1^. While age-standardized mortality rates have declined steadily in many regions since the late twentieth century, absolute IHD deaths continue to rise, driven by population growth and demographic aging^2^. Contemporary global analyses extending into 2025 have sharpened attention on regional heterogeneity, sex differences, and age-specific trends that challenge assumptions of uniform progress^3,4^.

A critical and emerging dimension of this global burden is the rise of early-onset IHD^5^. Recent evidence from the Global Burden of Disease (GBD) 2021 study, published in 2025, demonstrates an increasing global prevalence and incidence of IHD among young and middle-aged populations (15-49 years)^6^. While mortality rates in this specific age group have shown a slight downward trend, the highest burden remains concentrated in countries with low-middle socio-demographic indices^7,8^. This demographic shift is attributed mainly to dietary risks and gendered disparities, with men in this age bracket bearing a significantly higher burden^9,10^.

Recent work using the World Health Organization (WHO) mortality database and updated GBD frameworks has refined estimates of IHD mortality while exposing these persistent inequities^11,12^. Meanwhile, advances in epidemiologic modeling, improved cause-of-death attribution, and expanding data availability from low-resource settings are reshaping how global cardiovascular risk is understood^13^. This Perspective extends prior GBD and Lancet Commission^11^ analyses in three keyways.

**Figure 1:**
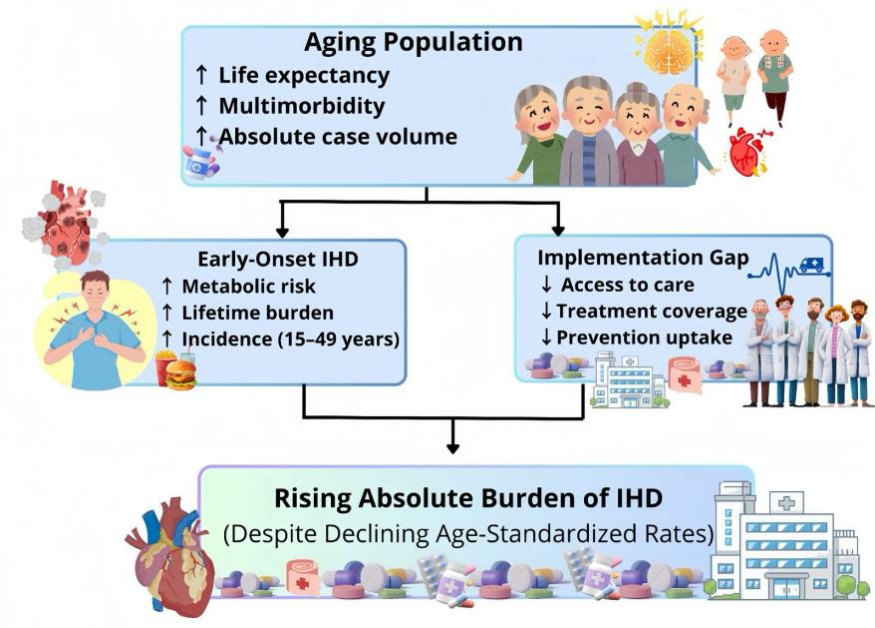
Graphical Abstract: A Unified Framework for the Persistent Global Burden of Ischemic Heart Disease The continued rise in the absolute burden of ischemic heart disease (IHD) reflects the interaction of three converging forces. Population aging increases the total number of individuals at risk and living with disease. Simultaneously, rising early-onset IHD expands lifetime exposure to cardiovascular risk and disease burden. These trends are compounded by persistent gaps in implementation, including limited access to prevention, delayed diagnosis, and suboptimal treatment coverage, particularly in low- and middle-income settings. Together, these factors explain the paradox of increasing absolute IHD burden despite declining age-standardized mortality rates.

First, it conceptualizes the rise of early-onset ischemic heart disease as a distinct phase within the epidemiologic transition, rather than a deviation from expected trends. Second, it integrates demographic aging, early-onset disease, and health system constraints into a unified framework to explain the persistence of the global burden despite declining age-standardized rates. Third, it links these epidemiologic patterns to specific, scalable implementation strategies, moving beyond descriptive analyses toward actionable pathways for reducing inequities (**Figure 1**, **Table 1**).

**Table 1:** Global Ischemic Heart Disease Mortality: Key Epidemiologic Patterns, Disparities, and Implications

Domain	Contemporary Evidence	Implications for Research and Policy
Overall Trends	Global age-standardized IHD mortality has declined, while absolute deaths continue to rise due to population aging.	Report crude and age-standardized rates concurrently to inform planning.
Regional Variation	Sustained mortality declines in high-income regions; stagnation or increases in parts of Africa and Latin America.	Prioritize equitable investment in cardiovascular HS.
Risk Factor Shifts	Declines in smoking and cholesterol are offset by rising obesity and diabetes prevalence.	Reorient prevention toward metabolic risk and food systems.
Health System (HS) Performance	Access to acute coronary care and secondary prevention strongly predicts survival.	Expand essential medicines, emergency systems, and continuity of care.
Sex Differences	Higher mortality in men; under-recognition and undertreatment in women persist.	Implement sex-specific diagnostic and prevention strategies.
Age-Specific Burden	Mortality is highest among adults ≥75 years; aging drives absolute burden.	Integrate IHD management with geriatric and multimorbidity care.
Data Limitations	Under-reporting and misclassification are common in low-resource settings.	Strengthen civil registration and cause-of-death attribution.
Socioeconomic Drivers	Persistent gradients independent of traditional risk factors.	Address social and commercial determinants of cardiovascular health.
Research Gaps	Limited translation of epidemiologic insights into implementation	Expand implementation science and policy-focused research.
Future Outlook	IHD burden is increasingly concentrated in LMICs and among older adults.	Develop equity-centered, life-course cardiovascular strategies.

## **2.** Advances in Global Epidemiology and Measurement

### 2.1. Refinement of Mortality Surveillance

Recent methodological advances have significantly strengthened the accuracy and reliability of IHD mortality estimation^14^. Updated modeling approaches, aligned with the GBD framework, incorporate sophisticated redistribution algorithms to address the ill-defined nature of cardiovascular deaths^15^. These models leverage Bayesian hierarchical frameworks to effectively address data sparsity in low-income regions, providing a more robust picture of the disease in underserved populations. Such refinements have successfully narrowed uncertainty intervals while confirming broad global trends, specifically the persistent decline in age-standardized IHD mortality alongside a marked and troubling regional divergence^16,17^.

The expanded use of join point regression and age-period-cohort analyses has further enabled more granular detection of critical inflection points in mortality trajectories^18^. These tools allow researchers to pinpoint exactly when and where prevention strategies have succeeded or plateaued. Notably, several landmark 2025 studies demonstrate that crude mortality rates are increasingly diverging from age-standardized trends. This divergence underscores the growing and dominant influence of population aging on the absolute IHD burden, signaling that health systems must prepare for a higher volume of patients even as individual risk profiles appear to improve^19^. While prior GBD analyses have characterized these trends in detail, they have largely treated early-onset and late-life IHD as part of a continuous spectrum. The current synthesis suggests that these patterns may instead reflect distinct epidemiologic dynamics with different underlying drivers and policy implications.

### 2.2. Integration of Risk Attribution

Another notable advance in the field is the sophisticated integration of cause-specific mortality with granular risk factor attribution. Contemporary analyses now more precisely quantify the proportion of IHD deaths attributable to hypertension, dyslipidemia, diabetes, smoking, dietary risks, and ambient air pollution. These studies reinforce that reductions in tobacco use and improvements in lipid levels account for a substantial fraction of the mortality declines observed in high-income countries^20-22^.

**Figure 2:**
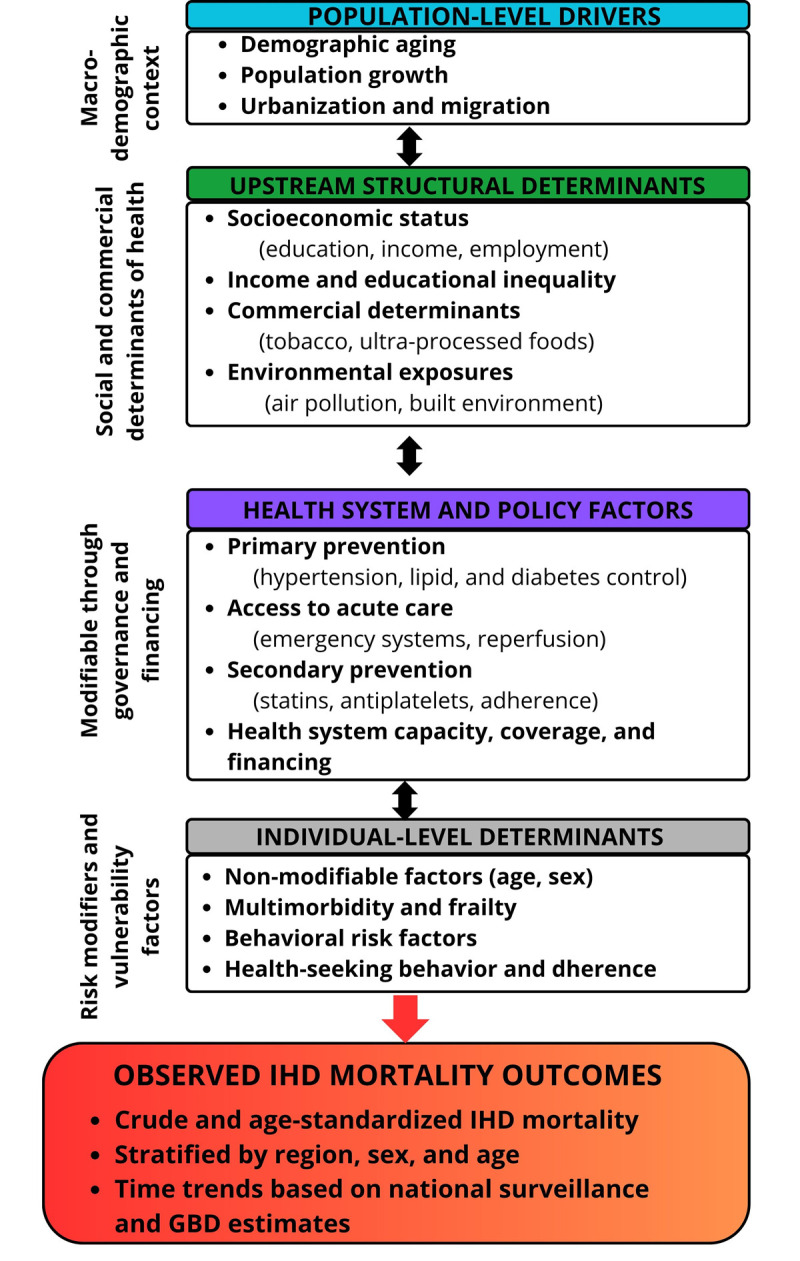
Multilevel Determinants of Global Ischemic Heart Disease Mortality Trends This conceptual framework illustrates the interacting determinants shaping global IHD mortality trends over the past two decades. Population-level drivers such as demographic aging, population growth, and urbanization interact with upstream structural determinants, including socioeconomic conditions, commercial influences, and environmental exposures. These factors converge with health system capacity, including primary prevention, acute coronary care, and secondary prevention, to influence individual-level risk modifiers related to sex, age, behavior, and comorbidity burden. The combined effects of these domains determine observed regional, sex-specific, and age-specific differences in crude and age-standardized IHD mortality rates worldwide. The figure highlights that sustained reductions in IHD mortality require coordinated, multisectoral action across societal, policy, and health system domains, rather than reliance on biomedical advances alone.

In contrast to the trends observed in high-income countries, rising metabolic risk factors such as obesity and diabetes now dominate the epidemiological landscape in many low and middle-income regions^23^. Evidence from the GBD framework suggests that dietary risks have emerged as the leading risk factor for early onset IHD, among populations aged 15 to 49 years^6^. This demographic shift indicates that the cardiovascular transition is no longer limited to the management of traditional lifestyle choices but is increasingly shaped by ambient environmental exposures and the commercial determinants of global food systems. Consequently, a purely biomedical approach is insufficient to address a burden that is increasingly rooted in the socioeconomic and policy environments of rapidly urbanizing regions (**Figure 2**).

## 3. Regional Inequities and Health System Determinants

Despite global progress, regional inequities in cardiovascular health remain pronounced^24^. According to Roth et al. (2025), high-income regions, including Western Europe, North America, and parts of Oceania, continue to experience sustained declines in both crude and age-standardized IHD mortality^17^. In contrast, several African regions and parts of Central and South America show stable or increasing crude mortality, even when age-standardized rates have plateaued. Recent data indicates that the absolute number of IHD deaths is rising in these regions, mainly because progress in reducing mortality rates has not kept pace with rapid population growth and demographic aging^25^.

Emerging evidence emphasizes that these disparities are not solely attributable to the prevalence of traditional risk factors. As reported by Rodriguez et al. (2025), health system capacity, which includes access to acute coronary care, the availability of essential medications, and the continuity of secondary prevention, exerts a dominant influence on survival after IHD events^26^. Furthermore, as indicated by Freihat et al. (2025), the rising burden of metabolic risk factors in low and middle-income countries remains a significant barrier to achieving the declines observed in wealthier nations^27^ .

While the landmark studies by Rodriguez et al. (2025) and Freihat et al. (2025) provide unparalleled statistical clarity, they also expose a critical limitation in contemporary cardiovascular epidemiology: the over-reliance on aggregated data that often masks the lived reality of vulnerable subgroups^17,26,27^. The persistent focus on broad regional trends can overlook that the cardiovascular transition is not linear but fragmented, with successes in high-income regions often built on infrastructures that are currently unscalable in low-resource settings. Evidence from recent studies suggests that emphasizing risk factor prevalence alone is insufficient; it is also necessary to assess how systemic failures in health delivery disproportionately penalize individuals based on their age and sex^26,27^. Specifically, the transition to sex and age-specific patterns reveals that our global strategies are often blind to the rising burden in younger populations and the diagnostic inertia facing older women. These patterns underscore the need for a greater focus on implementation science to understand why survival gains observed in high-income settings have not been consistently replicated across regions (**Figure 2**).

## 4. Sex and Age-Specific Patterns: Beyond Biology

### 4.1. Persistent Sex Differences and the Burden of the Young

We propose that the increasing burden of early-onset IHD^28^ represents an emergent and under-recognized phase of the epidemiologic transition, characterized by metabolic risk accumulation at younger ages and distinct from traditional late-life cardiovascular disease patterns. While men continue to experience higher IHD mortality rates than women across most regions, recent data reveal narrowing gaps in some populations^29,30^. However, evidence indicates that women, particularly older women, remain vulnerable to delayed diagnosis, atypical presentation, and lower use of evidence-based therapies^31^. These sex differences in outcomes reflect not only biological variation but also gendered patterns of care and health-seeking behavior.

As suggested by the 2025 analysis from Song et al**.**, men in this younger demographic bear a significantly higher burden of the disease, with dietary risks emerging as the primary factor driving early onset IHD^6^. However, it is important to recognize that this rise in early-onset cases constitutes an epidemiological challenge distinct from the burden observed in older cohorts. This demographic shift indicates that while global health strategies are beginning to manage the traditional risks associated with older age, we are failing to prevent metabolic acceleration among younger and middle-aged adults. This dual burden necessitates a strategic pivot, as the healthcare system must now simultaneously address preventable premature mortality in the young while managing the inevitable complexities of a rapidly growing aging population.

### 4.2. Aging Populations and the Absolute Burden

As life expectancy increases globally, age remains the strongest and most immutable determinant of IHD mortality^32^. Recent evidence shows that the absolute number of deaths among individuals aged 75 years and older continues to rise, even in regions where age-standardized rates are steadily declining^33^. This paradox highlights limitations in current health policy, where gains in primary prevention are offset by the increasing absolute number of individuals at risk due to population aging. Recent global analyses indicate that demographic change is now a driver of IHD burden, even in settings with declining age-standardized rates. However, the challenge of aging is not merely a matter of population size; it is a clinical complexity that the current biomedical model is ill-equipped to address^34^. Recent analyses highlight that failing to tackle multimorbidity, frailty, and social isolation among older adults will limit further gains in global mortality^35^. The traditional focus on single disease management is increasingly obsolete in the face of elderly patients who present with a constellation of competing health interests. The persistence of the global IHD burden reflects the interaction of three major forces: demographic aging, rising early-onset disease, and gaps in implementation of prevention and care (**Figure 2**).

This transition from acute IHD management to the care of the complex elderly reveals a profound gap in our current research and policy frameworks^36^. These observations suggest a potential imbalance in health system priorities, where investments in advanced interventions may not be matched by comparable development of integrated care models for older adults with multimorbidity^37^. Unless we shift toward a life course approach that integrates IHD management with geriatric care, the demographic transition will continue to outpace our medical successes. This clinical imperative to harmonize geriatric care with cardiovascular management is not merely a medical necessity but a foundational requirement for the next era of global health policy^38^. To move from descriptive epidemiology to tangible mortality reduction, we must prioritize the following four pillars of systemic reform^39^. Taken together, the coexistence of rising early-onset disease and an expanding aging population suggests a dual-burden model of IHD, in which health systems must simultaneously address premature disease and complex late-life multimorbidity. This duality is not fully captured in current epidemiologic frameworks.

## 5. Limitations of Current Epidemiological Models

This perspective relies primarily on aggregate estimates derived from the GBD framework and similar epidemiological models. Although GBD incorporates advanced methods to address incomplete data, including the redistribution of ill-defined causes of death, these approaches remain constrained by the variable quality of primary vital statistics^40-42^. The precision of global cardiovascular estimates is affected by limitations in data infrastructure, particularly in low- and middle-income countries, where civil registration systems remain incomplete or inconsistent^43^. In many settings, this results in underreporting and misclassification of causes of death, which can influence the reliability of mortality estimates^44^.In addition, the use of heterogeneous case ascertainment methods, including verbal autopsies, introduces variability in diagnostic classification, particularly for acute cardiovascular events such as sudden cardiac death^45^. The lack of large-scale, nationally representative cohort data with long-term follow-up further limits the ability to capture local epidemiological patterns, often requiring reliance on modeled extrapolations from comparable populations^46^. These methodological constraints limit causal inference, as aggregated observational estimates may not fully account for unmeasured confounding or broader health system disruptions. This is particularly relevant in contexts affected by external shocks, such as the COVID-19 pandemic, which altered both mortality patterns and healthcare access^47^ .Taken together, these factors contribute to uncertainty in global cardiovascular estimates and should be considered when translating epidemiological data into context-specific health policies.

## 6. Future Directions and Policy Imperatives

Unlike prior global strategies that primarily emphasize risk factor control or health system strengthening in isolation, this Perspective integrates epidemiologic trends with implementation science to identify where and why existing interventions fail to translate into population-level impact. The conceptual framework (**Figure 1**) highlights where scalable interventions can target each of these interacting drivers to reduce absolute burden.

### 6.1. Strengthening Global Surveillance and Data Systems

The foundation of any effective public health response is accurate and granular data. Strengthening civil registration and vital statistics systems must be prioritized, particularly in low-resource settings where underreporting and misclassification are most prevalent. Advances in analytical approaches indicate that the integration of Bayesian hierarchical frameworks and refined redistribution algorithms is essential to narrowing uncertainty intervals in regions with sparse data^48^. Beyond traditional mortality metrics, future research should incorporate longitudinal risk exposure and health system performance metrics to provide a more nuanced understanding of mortality drivers.

### 6.2. Achieving Equitable Prevention and Clinical Care

To achieve meaningful reductions in IHD mortality, we must expand primary prevention efforts, including aggressive blood pressure control, lipid management, and tobacco regulation. The implementation of tobacco taxation represents a cost-effective population-level strategy to reduce cardiovascular risk^49^. Evidence from modelling studies suggests that sustained tax increases are associated with consistent declines in tobacco consumption, with some analyses reporting reductions of up to 30%^50^. These changes translate into measurable health benefits at the population level, including reductions in overall mortality and fewer hospitalizations related to cardiovascular disease^51^.

These efforts must be accompanied by improvements in acute coronary care and long-term secondary prevention to ensure that survival gains are not limited to high-income regions^52^. As highlighted by Roth et al. (2025), our future strategies must be equity-centered and cover the entire life course, ensuring that the cardiovascular transition does not leave low- and middle-income countries^17^. As previously noted, dietary risks have emerged as a leading driver of early-onset IHD globally. To address this structural challenge, equitable prevention efforts must incorporate universal metabolic risk reduction strategies. The legislative elimination of industrial trans fatty acids (iTFA), through mandatory national limits or complete bans on partially hydrogenated oils, reduces dietary exposure more effectively than voluntary industry reformulations^53^. The public health impact of these upstream regulations is supported by modelling evidence suggesting that such policies can prevent a substantial number of ischemic events while generating meaningful healthcare savings, particularly in LMICs^54^ .

The impact of these interventions can be enhanced when combined with community-based strategies. Integrating policy measures with local health promotion has been associated with improvements in dietary practices and cardiovascular risk awareness^55^. A similar pattern is observed with salt reduction policies. Structural interventions, including reformulation of processed foods and the use of low-sodium substitutes, have been consistently associated with reductions in population-level blood pressure^56^. These changes translate into measurable declines in stroke and ischemic heart disease outcomes^57^.

Importantly, these regulatory approaches do not depend on individual behavioral change and tend to have a broader and more sustained impact across populations. They may also disproportionately benefit lower socioeconomic groups, where dietary risks are often highest, thereby contributing to reductions in health inequalities^58^.

### 6.3. Implementing Sex and Age Responsive Health Policies

The persistent disparities in care for women and the rising absolute burden among the elderly require tailored policy interventions. A shift beyond purely biological observations is needed to address gendered patterns of care and the complex needs of older adults facing multimorbidity and frailty. Emerging evidence suggests that implementing sex-specific diagnostic and preventive strategies will be essential to closing the survival gap^59,60^. Additionally, addressing the growing absolute burden of IHD among older adults will require a paradigm shift that integrates cardiovascular management with comprehensive geriatric assessment^61^.

Recent subnational analyses in Mexico, based on national mortality data, show that over one-third of all deaths are attributable to IHD and diabetes, with a disproportionate burden among older adults and marked regional clustering, reflecting structural gaps in prevention and care rather than demographic change alone^62^.

### 6.4. Prioritizing Implementation Science and Policy Reform

A major challenge in the current era is translating proven biomedical interventions into diverse real-world contexts, particularly in low- and middle-income countries^63^. Implementation science is increasingly recognized as central focus of cardiovascular research to identify and overcome the structural barriers to care delivery^64,65^. As noted earlier, limited health system capacity in many low- and middle-income countries continues to restrict survival gains^66^. Addressing these gaps requires shifting part of cardiovascular care beyond traditional clinical settings. Community-based and home-based screening programs have been shown to improve early detection, particularly among individuals who remain asymptomatic and outside formal healthcare systems^55^. Task-sharing represents a practical strategy to expand access by redistributing selected clinical responsibilities to non-physician health workers^67^. Complementing task-sharing approaches, several scalable, evidence-based interventions have demonstrated potential to reduce cardiovascular risk at the population level. Randomized trials, including HOPE-4 and TIPS-3, have demonstrated that fixed-dose combination strategies improve treatment adherence and achieve meaningful reductions in blood pressure and low-density lipoprotein cholesterol^68-72^. In addition, pragmatic studies such as the PolyIran trial have shown significant reductions in major cardiovascular events in real-world settings^73^. These strategies may be particularly effective in low-resource settings, where simplified treatment regimens can help overcome barriers related to access and continuity of care^74^. At the health system level, the World Health Organization Package of Essential Noncommunicable Disease Interventions (WHO PEN) provides a standardized framework for delivering cost-effective cardiovascular prevention and management in primary care settings^75^. WHO PEN emphasizes risk-based screening, standardized treatment protocols, and task-sharing approaches, and has shown feasibility in multiple low- and middle-income countries^76^. Despite their proven effectiveness and scalability, the adoption of these interventions remains uneven, reflecting broader challenges in implementation and health system capacity^77^. In some settings, this approach has extended to diagnostic capacity, where the use of point-of-care ultrasound by frontline providers can support earlier decision-making without compromising care quality^78^. These models also benefit from the close relationship between community health workers and patients, which has been associated with better adherence to treatment and improved blood pressure control^79,80^. For these strategies to be sustainable, they must be supported by clear policy frameworks that ensure adequate training, supervision, and remuneration of the workforce^5,8,9,40,45,49^. Together, these approaches offer a feasible pathway to expand equitable cardiovascular care in resource-constrained settings. Ultimately, reducing global IHD mortality requires a coordinated effort that aligns biomedical innovation with structural social and policy reforms to address the commercial and environmental determinants of health^7^. Recent analyses suggest that the largest near-term reductions in IHD mortality may derive from improved delivery and uptake of existing evidence-based therapies, particularly in low-resource settings. These implementation-ready strategies suggest that the persistence of the global IHD burden is driven less by a lack of effective interventions than by gaps in their equitable delivery.

## 7. Conclusion: From Metrics to Mortality

This Perspective advances a unifying interpretation of global IHD trends by linking three converging forces, demographic aging, the rise of early-onset disease, and persistent gaps in implementation, into a single explanatory framework for the continued growth in absolute burden. This evolving epidemiologic transition underscores the disconnect between declining age-standardized mortality and the rising absolute burden of ischemic heart disease worldwide. A critical reassessment is needed of a global framework that celebrates declining age-standardized rates in high-income settings while overlooking the rising absolute burden of IHD among the world’s most vulnerable populations. This requires prioritizing measurable improvements in delivery systems, including expansion of primary care–based cardiovascular screening, equitable access to timely reperfusion and acute cardiac care, and systematic implementation of secondary prevention programs. Proven, cost-effective strategies, such as polypill-based prevention, task-sharing with non-physician health workers, and integration of cardiovascular care into universal health coverage frameworks, should be scaled globally. Emerging evidence indicates that the persistent diagnostic inertia affecting women and older adults is not a biological inevitability, but rather a consequence of gaps in medical education and healthcare policy^59^. We argue that the traditional biomedical focus on single-disease management is increasingly obsolete in a world defined by aging, multimorbidity, and social isolation. The demographic momentum of a rapidly aging global population will continue to drive increases in absolute mortality unless a fundamental shift is made toward a life-course approach that integrates IHD prevention with geriatric and social care. Ultimately, the true measure of success will not be the precision of our mortality estimates, but the equity of health outcomes. A coordinated global effort is required to align biomedical innovation with health system strengthening, including regulatory policies targeting the commercial determinants of health, financing mechanisms to expand preventive care, and accountability metrics focused on equitable delivery of evidence-based interventions. To achieve a truly equitable reduction in the global IHD burden, there is increasing recognition that approaches focused solely on individual risk factors may be insufficient without addressing broader structural determinants of health, including access to care and socioeconomic conditions. Achieving more equitable global outcomes will depend on expanding access to proven interventions across diverse health system contexts. By moving beyond descriptive epidemiology, this framework provides a basis for aligning measurement, prevention, and health system reform.

## Acknowledgements

HACF, EPC, GB and EEJG are members of the Comité Científico de Salud of the Servicios de Salud de Oaxaca (SSO), Oaxaca, Mexico. HACF, MTHH, LPCM, EPC and EEJG are members of the Comité Oaxaqueño de Trombosis, Hemostasia y Endotelio (COTHE) of the SSO, Oaxaca, Mexico. This review received no specific grant from any funding agency in the public, commercial, or not-for-profit sectors. We have not been paid by a pharmaceutical company or any other agency to write this paper. We were not precluded from accessing data in the study, and we all accept responsibility for the decision to submit for publication. The authors declare that all data supporting the findings of this study are presented in the paper.

## Generative AI Statement

The authors declare that no artificial intelligence (AI) - assisted technologies (such as large language models, chatbots, or image creators) were used in the preparation of this manuscript.

## Author contributions

NSNS, ZMG, HACF, MTHH and VS led the conceptualization, methodology, and writing of the original draft. NSNS, ZMG, SMC, MMSO, CZO, LPCM, EEJG, VS, GB, EPC, HACF and MTHH assisted with methodology and data validation. NSNS, ZMG, EEJG, HACF, EPC, MTHH and VS contributed to writing the original draft and reviewing and editing the subsequent drafts. All authors contributed equally to revisions and review of the revised manuscript for resubmission.
